# Supplementary material for: Spelling interface using intracortical signals in a completely locked-in patient enabled via auditory neurofeedback training
Source: Nat Commun. 2022 Mar 22;13:1236. doi: 10.1038/s41467-022-28859-8 (PMC8941070; doi:10.1038/s41467-022-28859-8)
Supplement: Supplementary file 1 — Supplementary information file [file 41467_2022_28859_MOESM1_ESM.pdf]

Spelling Interface using Intracortical Signals in a Completely Locked-In Patient enabled via Auditory Neurofeedback Training

Ujwal Chaudhary[1+*], Ioannis Vlachos[2+], Jonas B. Zimmermann[2+*], Arnau Espinosa[2], Alessandro Tonin[1,2], Andres Jaramillo-Gonzalez[2], Majid Khalili-Ardali[2], Helge Topka[3], Jens Lehmberg[4], Gerhard M. Friehs[5], Alain Woodtli[2], John P. Donoghue[6], and Niels Birbaumer[1*]

[1]Institute of Medical Psychology and Behavioral Neurobiology, University of Tübingen, Germany

[2]Wyss Center for Bio and Neuroengineering, Geneva, Switzerland

[3]Department of Neurology, Clinical Neurophysiology, Cognitive Neurology and Stroke Unit München Klinik Bogenhausen, Munich, Germany.

[4]Department of Neurosurgery, München Klinik Bogenhausen, Munich, Germany.

[5]Neurosurgery Department, European University, Nicosia, Cyprus.

[6]Carney Brain Institute, Brown University, Providence, USA.

[+]These authors contributed equally

*

chaudharyujwal@gmail.com;
or
jonas.zimmermann@wysscenter.ch;
or
niels.birbaumer@uni-tuebingen.de

Supplementary Figure S1: Audio-Neurofeedback Accuracy – a) Accuracy in all neurofeedback sessions from day 106 (first-time neurofeedback speller was attempted) to day 462. The accuracy of each neurofeedback block is represented as a dot. The red dots represent neurofeedback blocks preceding a speller session. b) and c) Accuracies of neurofeedback sessions on two different days as examples. The x-axis is the time in minutes, representing the time at which a particular NF session, denoted by a black dot, was performed on that particular day, and the y-axis is the accuracy in percent. The overall average feedback session accuracy was 72%, taking into account even initialisation sessions in which parameters were not yet optimised. Source data are provided as a Source Data file.

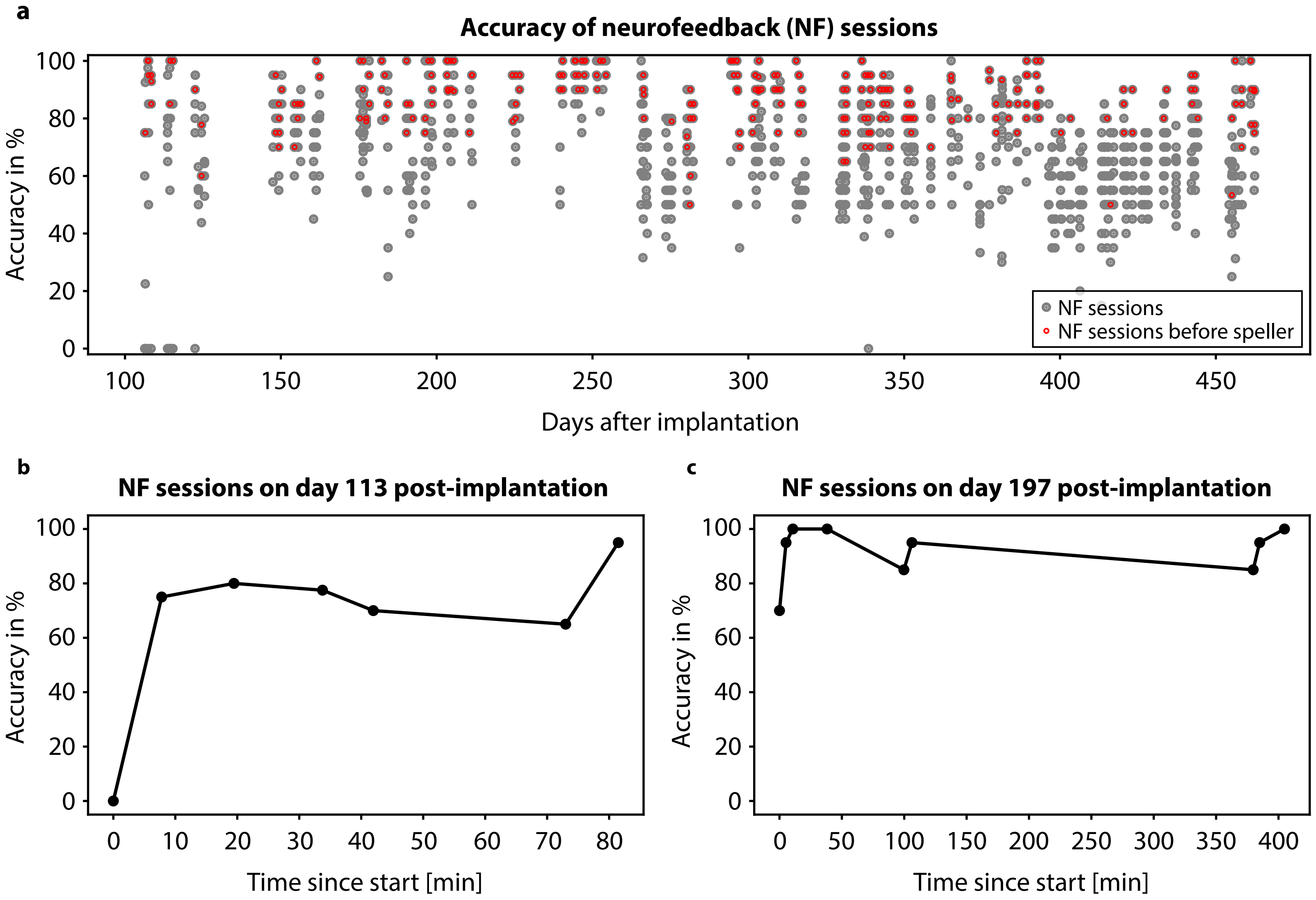

a
Accuracy of neurofeedback (NF) sessions
Accuracy in %
Days after implantation
NF sessions
NF sessions before speller
b
NF sessions on day 113 post-implantation
Accuracy in %
Time since start [min]
c
NF sessions on day 197 post-implantation
Accuracy in %
Time since start [min]

Supplementary Figure S2: State of the Utah array over time – a) Depicts the channels grouped by average firing rate. The dashed, thin solid, and thick solid trace represent the number of channels between 0.5 and 2 Hz firing rate, 2 and 10 Hz firing rate, and greater than 10 Hz firing rate, respectively, from day 26 to day 462 post-implantation. b) Depicts the channels grouped by electrode impedance. The dashed, thick solid and dotted trace represents the number of channels less than 100 kΩ, greater than 100 kΩ and less than 800 kΩ, and greater than 800 kΩ impedance, respectively, from day 26 to day 462 post-implantation. Source data are provided as a Source Data file.

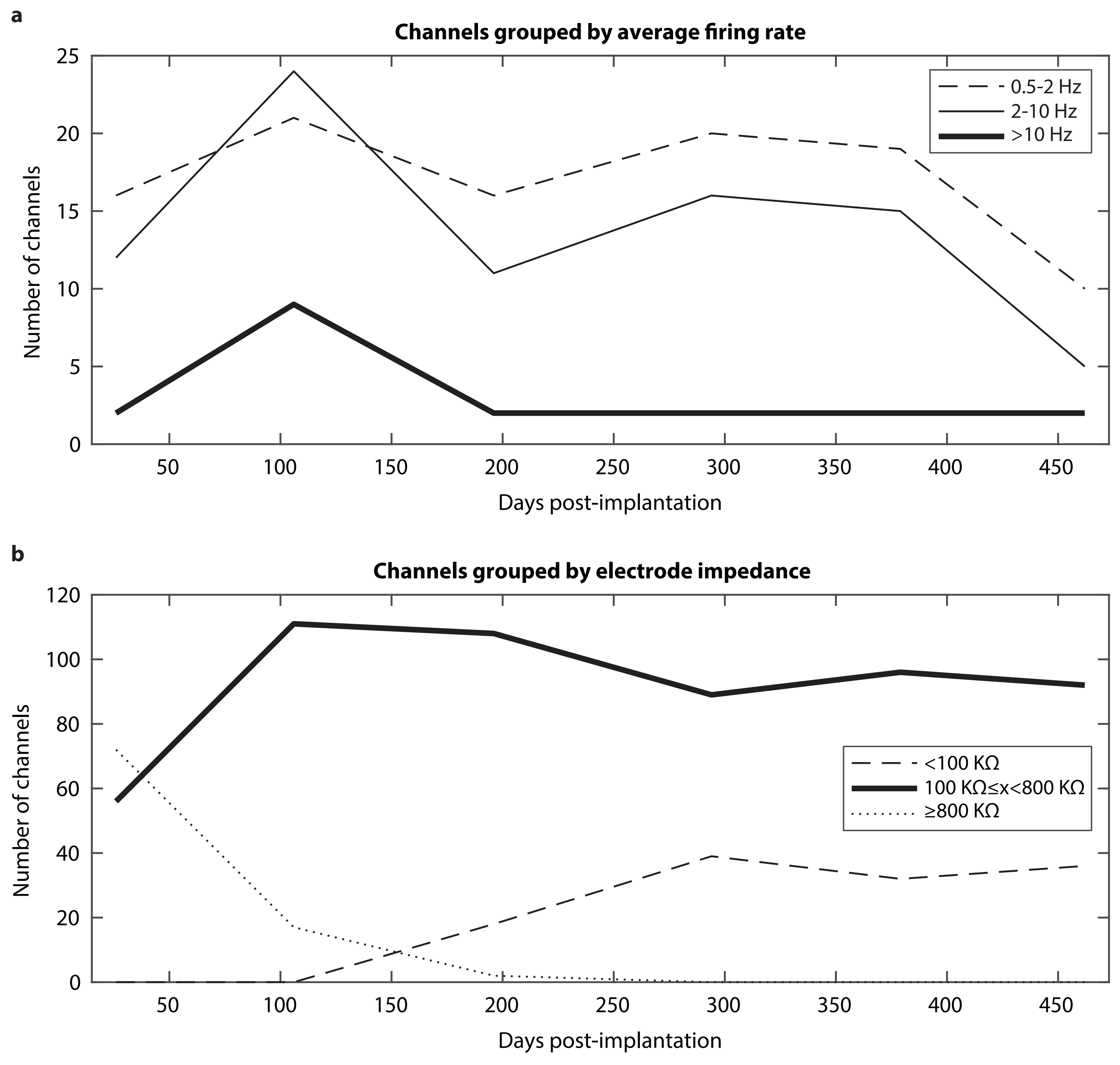
a
Channels grouped by average firing rate
0.5-2 Hz
2-10 Hz
>10 Hz
Number of channels
Days post-implantation
0
5
10
15
20
25
50
100
150
200
250
300
350
400
450
b
Channels grouped by electrode impedance
<100 KΩ
100 KΩ≤x<800 KΩ
≥800 KΩ
Number of channels
Days post-implantation
0
20
40
60
80
100
120
50
100
150
200
250
300
350
400
450

Supplementary Figure S3 – a) Channels used for speller control over time, sorted by frequency of use. The color of the marker indicates how many channels were used simultaneously during that particular speller block. One channel was used for control in 268 blocks, two were used in 56 blocks, three in 6, and four channels were used in 2 blocks. Each day is indicated by a tick mark. b) Channel use aggregated over recording days. c) Locations of channels shown in a and b on the array placed in superior frontal gyrus (SFG). Channels of the array in precentral gyrus (PreCG) were not used in any of the reported sessions due to lack of modulation. IHF: interhemispheric fissure, CS: central sulcus. Source data are provided as a Source Data file.

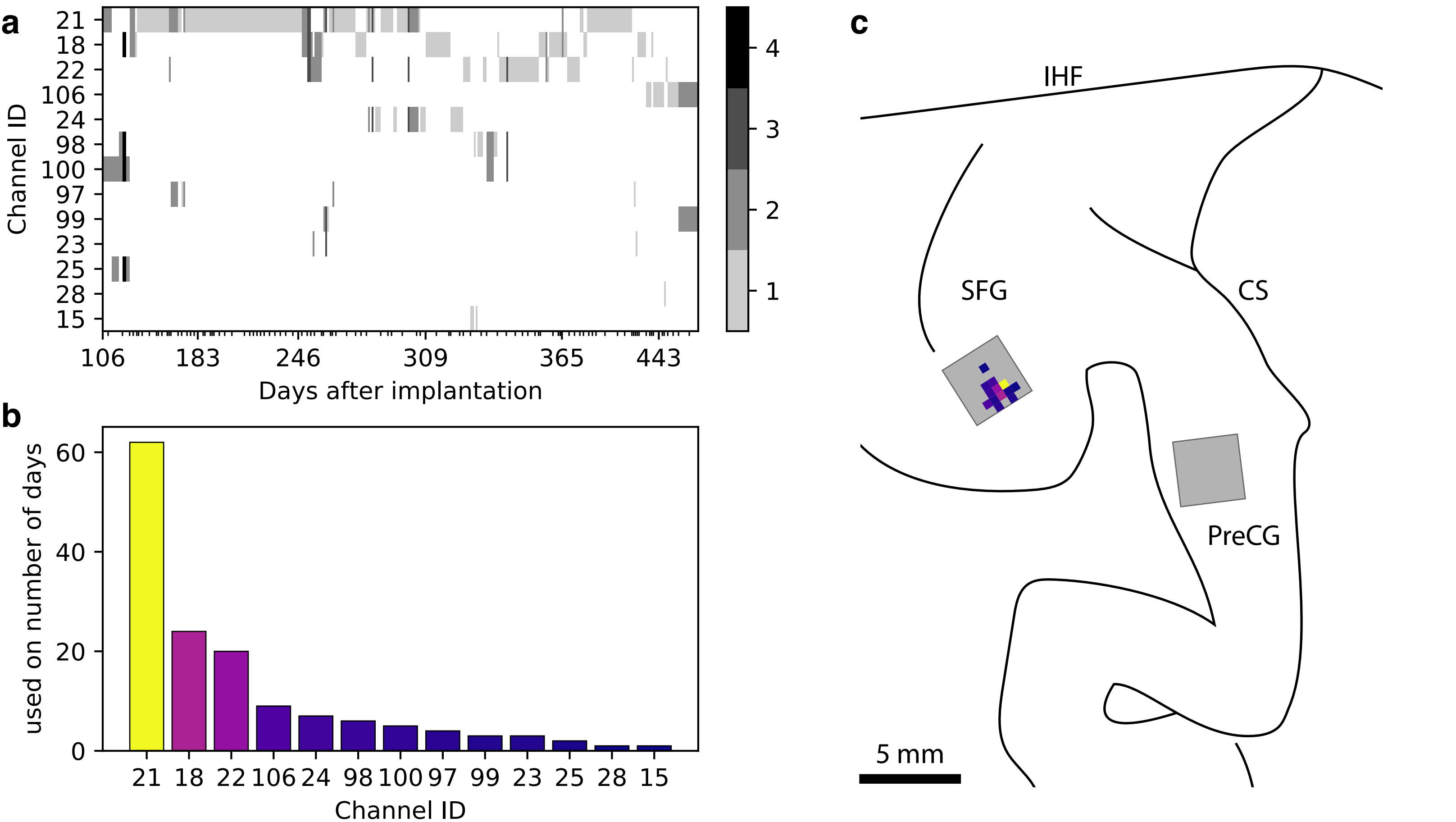
a
b
c
Channel ID
Days after implantation
106
183
246
309
365
443
21
18
22
106
24
98
100
97
99
23
25
28
15
1
2
3
4
used on number of days
0
20
40
60
Channel ID
IHF
SFG
CS
PreCG
5 mm

Supplementary Figure S4: Electrooculogram (EOG) features – evolution of the A) amplitude variance, B) mean amplitude, C) maximum amplitude, D) power in the band 0-2 Hz, and E) power in the band 2-4 Hz of the EOG signal for yes/no questions answered by the patient during the period March 2018 to November 2019. For each day, the vertical and horizontal EOG raw signal was filtered between 0.05 Hz and 40 Hz. Each yes and no trial was extracted and corrected by subtracting the mean of the trial's baseline. Finally, the corresponding feature of the EOG signal was calculated separately for each trial. The figure shows daily distributions of the extracted features, grouped by source (vertical and horizontal EOG) and condition (yes/no trials). Data that are outside 1.5 times the interquartile range are classified as outliers. Each box represents the data between the 25th and 75th percentile, median value is marked as horizontal line inside the box, whiskers show the remaining data excluding the outliers, which are represented as diamonds. For each day, a Mann-Whitney U-test was performed between yes and no results separately for the horizontal and vertical EOG, the results are represented by the significant *p*-values ($p<0.05$, no correction for multiple comparisons) shown above the distributions. The table in A) lists the date and the number of yes and no trials used for vertical and horizontal EOG. The x-axis represents the date of the sessions, and the y-axis represents the value of the corresponding feature. The vertical red line indicates the date of the implantation: 19 March 2019. The months of all the non-invasive sessions performed after implantation are highlighted in red on the x-axis. Source data are provided as a Source Data file.

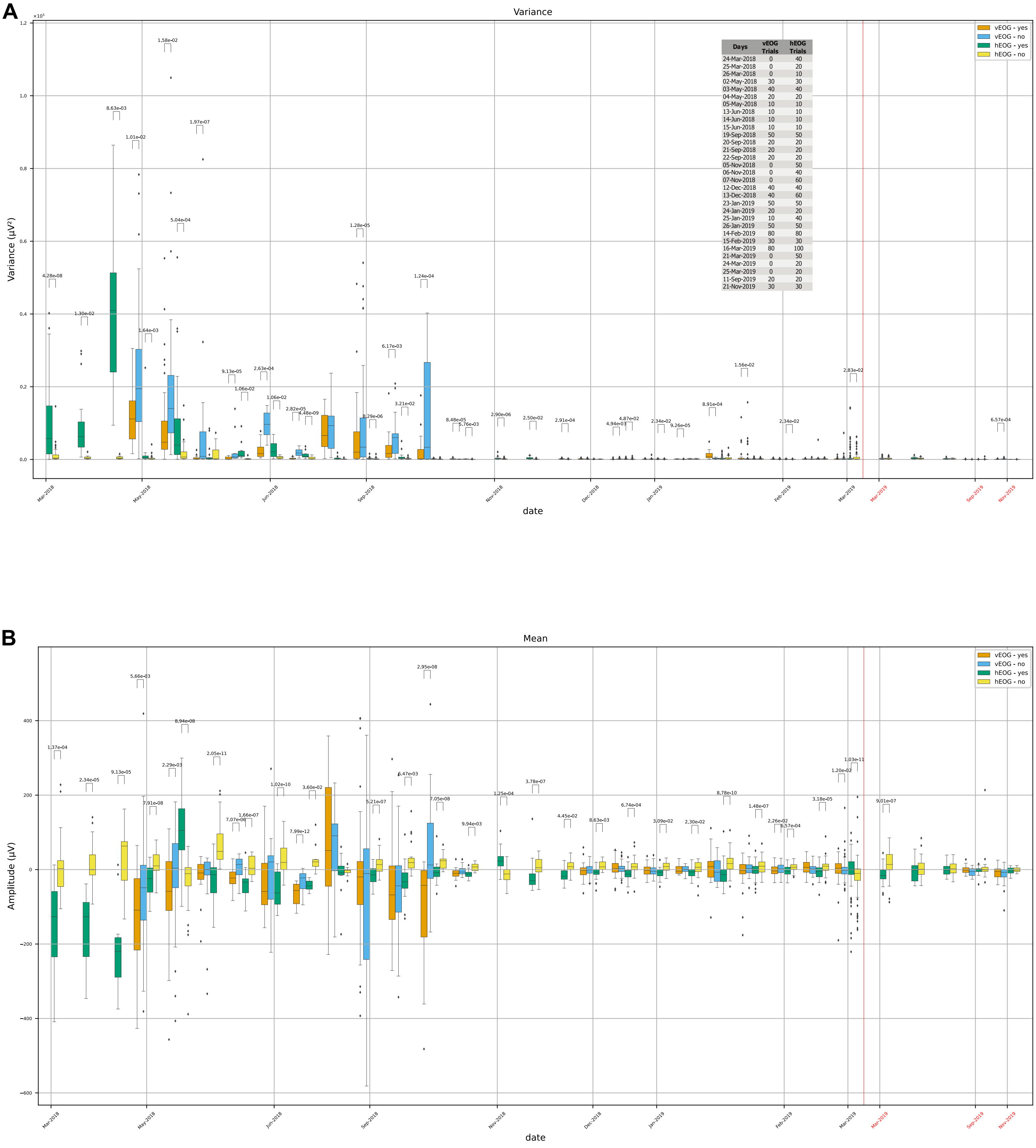
A
Variance
Variance (µV²)
date
vEOG - yes
vEOG - no
hEOG - yes
hEOG - no
Days vEOG Trials hEOG Trials
24-Mar-2018 0 40
25-Mar-2018 0 20
26-Mar-2018 0 10
02-May-2018 30 30
03-May-2018 40 40
04-May-2018 20 20
05-May-2018 10 10
13-Jun-2018 10 10
14-Jun-2018 10 10
15-Jun-2018 10 10
19-Sep-2018 50 50
20-Sep-2018 20 20
21-Sep-2018 20 20
22-Sep-2018 20 20
05-Nov-2018 0 50
06-Nov-2018 0 40
07-Nov-2018 0 60
12-Dec-2018 40 40
13-Dec-2018 40 60
23-Jan-2019 50 50
24-Jan-2019 20 20
25-Jan-2019 10 40
26-Jan-2019 50 50
14-Feb-2019 80 80
15-Feb-2019 30 30
16-Mar-2019 80 100
21-Mar-2019 0 50
24-Mar-2019 0 20
25-Mar-2019 0 20
11-Sep-2019 20 20
21-Nov-2019 30 30
B
Mean
Amplitude (µV)
date

C

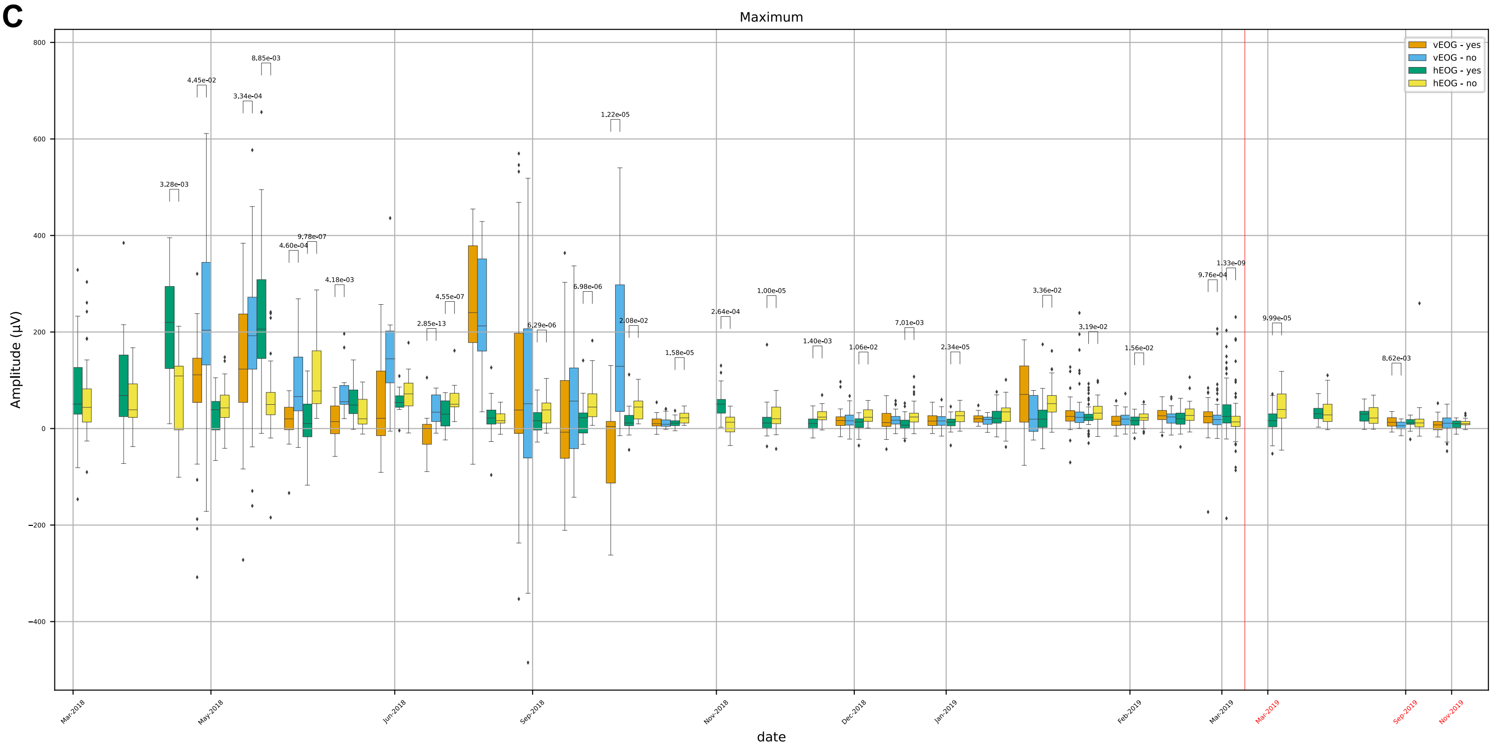
Maximum
Amplitude (µV)
date
vEOG - yes
vEOG - no
hEOG - yes
hEOG - no
Mar-2018
May-2018
Jun-2018
Sep-2018
Nov-2018
Dec-2018
Jan-2019
Feb-2019
Mar-2019
Mar-2019
Sep-2019
Nov-2019

D

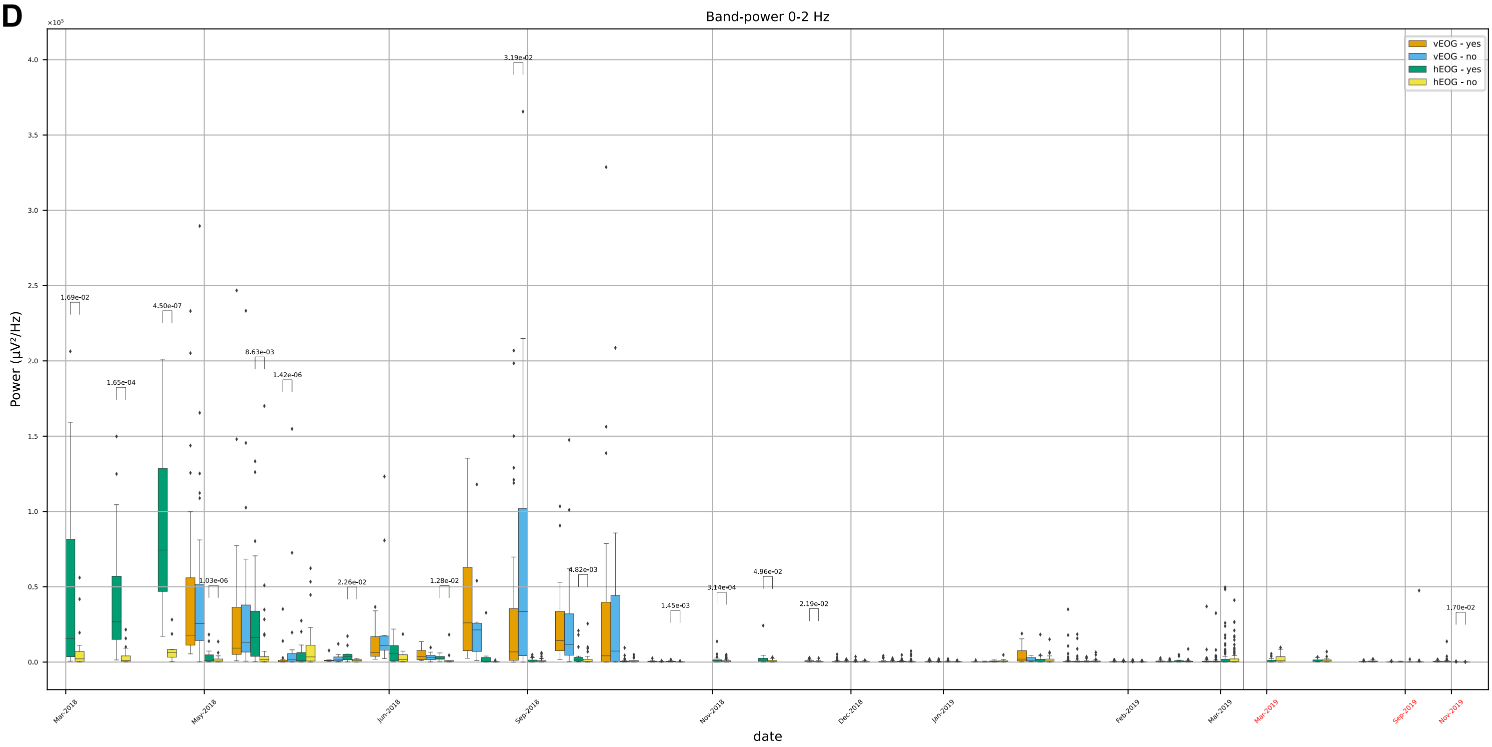
Band-power 0-2 Hz
Power (µV²/Hz)
date
vEOG - yes
vEOG - no
hEOG - yes
hEOG - no
Mar-2018
May-2018
Jun-2018
Sep-2018
Nov-2018
Dec-2018
Jan-2019
Feb-2019
Mar-2019
Mar-2019
Sep-2019
Nov-2019

E

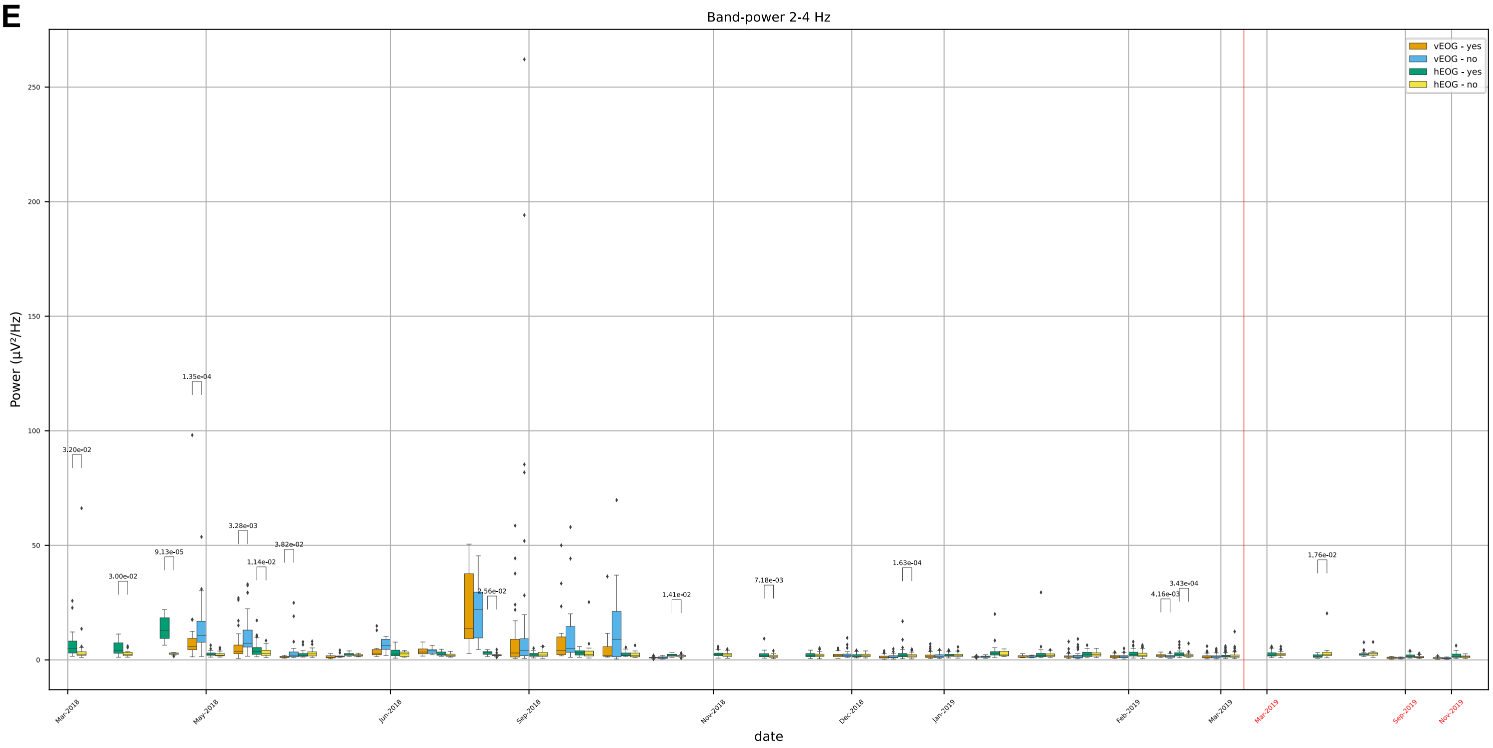
Band-power 2-4 Hz
vEOG - yes
vEOG - no
hEOG - yes
hEOG - no
Power (µV²/Hz)
0
50
100
150
200
250
3.20e-02
3.00e-02
9.13e-05
1.35e-04
3.28e-03
1.14e-02
3.82e-02
2.56e-02
1.41e-02
7.18e-03
1.63e-04
4.16e-03
3.43e-04
1.76e-02
Mar-2018
May-2018
Jun-2018
Sep-2018
Nov-2018
Dec-2018
Jan-2019
Feb-2019
Mar-2019
Mar-2019
Sep-2019
Nov-2019
date

Supplementary Note 1

EOG/EEG recordings and assessment of Completely Locked-In-State (CLIS)

Eye movements during the sessions were recorded with a 16 channel EEG amplifier (V-Amp DC, Brain Products, Germany) with Ag/AgCl electrodes. A total of four EOG electrodes were placed above and below the eyes and at the right and left canthi (EOGU) (up) and EOGD (down) for vertical eye movement, and EOGR (right) and EOGL (left) for horizontal eye movements. Usually 7 EEG-channels were recorded for all non-invasive hybrid BCI-eye-movement/EEG communication sessions and the sessions after implantation. All the channels were referenced to an electrode on the right mastoid and grounded to the electrode placed at FPz location on the scalp. Electrode impedances were kept below 10 kΩ. The sampling frequency was 500 Hz. Employing the non-invasive BCI-system, the patient was asked to explicitly move eyes for a “yes” and to not move the eyes for a “no” response. To further assess the ability of the patient to voluntarily control eye muscles after implantation, non-invasive EOG-BCI sessions were performed 3 days, 6 months, and 9 months after implantation.

Signal Analysis

The analysis of the electrooculographic (EOG) signal was performed for all the sessions in which the patient replied to yes/no questions requesting a known answer. Each session consists of 10 yes and 10 no questions (i.e., trials) whose answers are known by the patient (“Munich is in Germany” vs. “Munich is in Italy”). Each of these trials consists of the baseline (no question presented), the stimulus (presentation of the question), the response time (instruction: eye movement for saying “yes” and no movement for saying “no”), and feedback (feedback-tone to the patient to indicate the end of the response time). In total 109 sessions have been recorded. Two sessions that were interrupted during the experiment have been excluded, bringing the total number of sessions of this type to 107.
The sessions before the implantation (19th March 2019) were recorded using active electrodes, while after the implantation passive electrodes were used for the recordings to avoid interference with the implanted system. A vAMP amplifier (BrainProducts, Munich, Germany) with a sampling rate of 500 Hz was used. Horizontal EOG signals were recorded in all sessions as the difference of two electrodes placed close to the side of eye for horizontal eye movements, the vertical EOG signal was recorded on the right eye using the difference of two electrodes on the placed above and below the eye, except for 5 sessions that were recorded from the left eye and 38 sessions in which vertical EOG

signal was not recorded because of a small skin lesion. In all the sessions electrodes were grounded to FPz and referenced to the right mastoid.

Seven blocks (each block consists of 20 question – 10 with yes answers and 10 with no answers) that showed noise on the raw vertical EOG signal were excluded, bringing the total number of recorded vertical EOG sessions to 62.

Vertical and horizontal signals from each session were individually filtered between 0.05 Hz and 40 Hz with a FIR filter using the function pop_eegfiltnew from the Matlab toolbox EEGLAB with automatic order selection.

After filtering, from each trial of each session the signal was corrected subtracting the mean of the respective baseline. Then, from the baseline-corrected response, four features in the time domain and two features in the frequency domain were extracted. The extracted time domain features are:

- mean (Matlab function mean),
- variance (Matlab function var),
- maximum (Matlab function max),
- range of the amplitude (Matlab function max-min);

and the extracted frequency domain features are:

- power of the band between 0 Hz and 2 Hz (Matlab function bandpower),
- power of the band between 2 Hz and 4 Hz (Matlab function bandpower).

All the trials were grouped day-wise (usually multiple sessions belong to the same day). For these daily groups, a non-parametric Mann-Whitney U-test was performed separately for each feature to test for statistical differences between "yes" and "no" trials (Python function mannwhitneyu from the library scikit-learn).

The results of these analyses are shown in Supplementary Figure S4 separately for each feature. In the figures, for vertical and horizontal EOG, each daily group of "yes" and "no" trials is represented with its mean and standard error of the mean, while the *p*-value from the Mann-Whitney U-test is reported on the top of the bar only if it is below 0.05.
