# Supplementary material for: Spelling interface using intracortical signals in a completely locked-in patient enabled via auditory neurofeedback training
Source: Nat Commun. 2022 Mar 22;13:1236. doi: 10.1038/s41467-022-28859-8 (PMC8941070; doi:10.1038/s41467-022-28859-8)
Supplement: Supplementary file 3 — Description of Additional Supplementary Files [file 41467_2022_28859_MOESM3_ESM.pdf]

Description of Additional Supplementary Files

File Name: Supplementary Movie 1
Description: A video showing 7 trials of a typical neurofeedback block (day 114 at12:46 hours).

File Name: Supplementary Movie 2
Description: A 5 min 30 second video of free speller block performed by the patient on day 247. The speller block lasted for 151 minutes from 15:30 to 18:07 hours.
