# Supplementary material for: Spelling interface using intracortical signals in a completely locked-in patient enabled via auditory neurofeedback training
Source: Nat Commun. 2022 Mar 22;13:1236. doi: 10.1038/s41467-022-28859-8 (PMC8941070; doi:10.1038/s41467-022-28859-8)
Supplement: Supplementary file 6 — Reporting Summary [file 41467_2022_28859_MOESM6_ESM.pdf]

nature research

Corresponding author(s): DBPR. NCOMMS-20-22295C.

Last updated by author(s): Nov 5, 2021

Reporting Summary

Nature Research wishes to improve the reproducibility of the work that we publish. This form provides structure for consistency and transparency in reporting. For further information on Nature Research policies, see our Editorial Policies and the Editorial Policy Checklist.

Statistics

For all statistical analyses, confirm that the following items are present in the figure legend, table legend, main text, or Methods section.

n/a	Confirmed	
☐	☒	The exact sample size (*n*) for each experimental group/condition, given as a discrete number and unit of measurement
☐	☒	A statement on whether measurements were taken from distinct samples or whether the same sample was measured repeatedly
☐	☒	The statistical test(s) used AND whether they are one- or two-sided<br>*Only common tests should be described solely by name; describe more complex techniques in the Methods section.*
☒	☐	A description of all covariates tested
☒	☐	A description of any assumptions or corrections, such as tests of normality and adjustment for multiple comparisons
☐	☒	A full description of the statistical parameters including central tendency (e.g. means) or other basic estimates (e.g. regression coefficient) AND variation (e.g. standard deviation) or associated estimates of uncertainty (e.g. confidence intervals)
☐	☒	For null hypothesis testing, the test statistic (e.g. *F*, *t*, *r*) with confidence intervals, effect sizes, degrees of freedom and *P* value noted<br>*Give P values as exact values whenever suitable.*
☒	☐	For Bayesian analysis, information on the choice of priors and Markov chain Monte Carlo settings
☒	☐	For hierarchical and complex designs, identification of the appropriate level for tests and full reporting of outcomes
☒	☐	Estimates of effect sizes (e.g. Cohen's *d*, Pearson's *r*), indicating how they were calculated

Our web collection on statistics for biologists contains articles on many of the points above.

Software and code

Policy information about availability of computer code

Data collection	We used a modified version of the CereLink library (https://github.com/dashesy/CereLink) to collect data from the Blackrock Neural Signal Processor. Custom software written in Python (3.6) and C++11 was used to perform and control all BCI sessions. Both Python and C++ software with a list of requirements (including version numbers) is available at https://doi.org/10.12751/g-node.ihc6qn.
Data analysis	Analysis in this paper was written in Python, to process log files and spike rate data files as published with the data repository.

For manuscripts utilizing custom algorithms or software that are central to the research but not yet described in published literature, software must be made available to editors and reviewers. We strongly encourage code deposition in a community repository (e.g. GitHub). See the Nature Research guidelines for submitting code & software for further information.

Data

Policy information about availability of data

All manuscripts must include a data availability statement. This statement should provide the following information, where applicable:

- Accession codes, unique identifiers, or web links for publicly available datasets
- A list of figures that have associated raw data
- A description of any restrictions on data availability

The data upon which the findings in this paper are based (neural firing rates, event log files for data presented in figures 2, 3, 4, S1, S3; electrode impedances and spike event files for data presented in Fig S2) are available at DOI:10.12751/g-node.jdwmjd. The EOG data which figure S4 is based on is available at DOI:10.12751/g-node.ng4dfr. Source data are provided with this paper. The raw neural recordings can be made available upon reasonable request to J.B.Z., yet owing to the potential sensitivity of the data, an agreement between the researcher's institution and the Wyss Center is required to facilitate the sharing of these datasets. Requests will be responded to within one month.

Field-specific reporting

Please select the one below that is the best fit for your research. If you are not sure, read the appropriate sections before making your selection.

☒ Life sciences ☐ Behavioural & social sciences ☐ Ecological, evolutionary & environmental sciences

For a reference copy of the document with all sections, see nature.com/documents/nr-reporting-summary-flat.pdf

Life sciences study design

All studies must disclose on these points even when the disclosure is negative.

Sample size	This is a single-case study
Data exclusions	All the data acquired during the neurofeedback communication session performed by the patient from day 106 to day 462 are reported here.
Replication	The patient performed communication sessions on 107 days, of which 44 days were rated as intelligible. The neurofeedback paradigm was replicated in 1176 sessions.
Randomization	This is a single-case study, in which the participant received an implant. To validate the neurofeedback paradigm, sessions were performed in which pseudo-randomly chosen targets were presented, for the participant to match with his neural activity.
Blinding	Blinding is not relevant for this study because we are just reporting the observed result without performing any new analysis.

Reporting for specific materials, systems and methods

We require information from authors about some types of materials, experimental systems and methods used in many studies. Here, indicate whether each material, system or method listed is relevant to your study. If you are not sure if a list item applies to your research, read the appropriate section before selecting a response.

Materials & experimental systems

n/a	Involved in the study	
☒	☐	Antibodies
☒	☐	Eukaryotic cell lines
☒	☐	Palaeontology and archaeology
☒	☐	Animals and other organisms
☐	☒	Human research participants
☒	☐	Clinical data
☒	☐	Dual use research of concern

Methods

n/a	Involved in the study	
☒	☐	ChIP-seq
☒	☐	Flow cytometry
☒	☐	MRI-based neuroimaging

Human research participants

Policy information about studies involving human research participants

Population characteristics	The patient, born in 1985, was diagnosed with progressive muscle atrophy, a clinical variant of non-bulbar ALS, selectively affecting spinal motor neurons in August 2015. He lost verbal communication and the capability to walk by the end of 2015. He has been fed through a percutaneous endoscopic gastrostomy tube and artificially ventilated since July 2016 and is in home care. He started using the MyTobii eye-tracking-based assistive and augmentative communication (AAC) device in August 2016. From August 2017 onwards, he could not use the eye-tracker for communication because of his inability to fixate his gaze.
Recruitment	The patient anticipated complete loss of eye control and asked for a new communication system, which motivated the family to contact authors NB and UC for alternative approaches. Initial assessment sessions were performed in February 2018. The patient and family were also informed that non-invasive BCI-systems might stop functioning satisfactorily, and in particular, selection of letters might not be possible if he became completely locked-in (where no eye movements could be recorded reliably). In that case, implantation of an intracortical BCI-system using neural spike-based recordings might allow for voluntary communication. As the patient's ability to communicate via non-invasive BCI systems deteriorated, in June 2018, preparations for the implantation of an intracortical BCI system were initiated.
Ethics oversight	The medical procedure was approved by the Bundesinstitut für Arzneimittel und Medizinprodukte ("BfArM", The German Federal Institute for Drugs and Medical Devices). The study was declared as a Single Case Study and has received a special authorization ("Sonderzulassung") by BfArM, according to §11 of the German Medical Device Law ("Medizin-Produkte-Gesetz") on December 20, 2018, with Case Nr. 5640-S-036/18. The Ethical Committee of the Medical Faculty of the Technische Universität München Rechts der Isar provided support to the study on 19 Jan 2019, along with the explicit permission to publish on 17 February 2020.

Note that full information on the approval of the study protocol must also be provided in the manuscript.
